# Prognostic Performance of Albumin–Bilirubin Grade With Artificial Intelligence for Hepatocellular Carcinoma Treated With Transarterial Chemoembolization Combined With Sorafenib

**DOI:** 10.3389/fonc.2020.525461

**Published:** 2020-12-18

**Authors:** Bin-Yan Zhong, Zhi-Ping Yan, Jun-Hui Sun, Lei Zhang, Zhong-Heng Hou, Min-Jie Yang, Guan-Hui Zhou, Wan-Sheng Wang, Zhi Li, Peng Huang, Shen Zhang, Xiao-Li Zhu, Cai-Fang Ni

**Affiliations:** ^1^ Department of Interventional Radiology, The First Affiliated Hospital of Soochow University, Suzhou, China; ^2^ Department of Interventional Radiology, Zhongshan Hospital, Fudan University, Shanghai, China; ^3^ Shanghai Institution of Medical Imaging, Shanghai, China; ^4^ National Clinical Research Center for Interventional Medicine, Shanghai, China; ^5^ Hepatobiliary and Pancreatic Interventional Treatment Center, Division of Hepatobiliary and Pancreatic Surgery, The First Affiliated Hospital, Zhejiang University School of Medicine, Hangzhou, China

**Keywords:** hepatocellular carcinoma, albumin–bilirubin, artificial intelligence, nomogram, artificial neural network

## Abstract

**Purpose:**

To establish albumin-bilirubin (ALBI) grade-based and Child-Turcotte-Pugh (CTP) grade-based nomograms, as well as to develop an artificial neural network (ANN) model to compare the prognostic performance and discrimination of these two grades for hepatocellular carcinoma (HCC) treated with transarterial chemoembolization (TACE) combined with sorafenib as an initial treatment.

**Methods:**

This multicenter retrospective study included patients from three hospitals between January 2013 and August 2018. In the training cohort, independent risk factors associated with overall survival (OS) were identified by univariate and multivariate analyses. The nomograms and ANN were established and then validated in two validation cohorts.

**Results:**

A total of 504 patients (319, 61, and 124 patients from hospitals A, B, and C, respectively) were included. The median OS was 15.2, 26.9, and 14.8 months in the training cohort and validation cohorts 1 and 2, respectively (P = 0.218). In the training cohort, both ALBI grade and CTP grade were identified as independent risk factors. The ALBI grade-based and CTP grade-based nomograms were established separately and showed similar prognostic performance and discrimination when validated in the validation cohorts (C-index in validation cohort 1: 0.799 vs. 0.779, P = 0.762; in validation cohort 2: 0.700 vs. 0.693, P = 0.803). The ANN model showed that the ALBI grade had higher importance in survival prediction than the CTP grade.

**Conclusions:**

The ALBI grade and CTP grade have comparable prognostic performance for HCC patients treated with TACE combined with sorafenib. ALBI grades 1 and 2 have the potential to act as a stratification factor for clinical trials on the combination therapy of TACE and systemic therapy.

## Introduction

Due to its insidious onset, approximately 80% of patients with hepatocellular carcinoma (HCC) are first diagnosed with intermediate or advanced stage, which means that they are ineligible for curative therapies such as resection, transplantation, or ablation (1, 2). For these patients, transarterial chemoembolization (TACE) is the major recommendation according to several international guidelines if they have preserved liver function and good performance status (3–5). In real-world clinical practice, especially in Asia, TACE is also widely applied in intermediate and advanced stages (6). By embolizing targeted arteries of the lesions to achieve tumor control, TACE induces ischemic and hypoxic changes that lead to an increase in vascular endothelial growth factor (VEGF) and platelet-derived growth factor (PDGF) expression locally in the residual lesion(s), which theoretically promotes recurrence or progression (7–9). In addition, repeated TACE may lead to the deterioration of liver function, resulting in poor prognosis (10).

Sorafenib, a potent multikinase inhibitor that mainly targets VEGF receptors, RAF, and PDGF receptor, is the major treatment approach for advanced HCC (2, 11, 12). It has antiangiogenic and antiproliferative effects that inhibit tumor growth and disrupt tumor microvasculature (11, 12). Therefore, the combination of TACE and sorafenib should have synergistic action for unresectable HCC. A preclinical study identified that the combination of TACE and antiangiogenic therapy reduces tumor volume and vessel density and prolongs survival when compared to TACE monotherapy (13). Nevertheless, several randomized controlled trials (RCTs) demonstrated a negative benefit for such combination therapy (14–16). Apart from the study design, including the treatment duration of sorafenib and sequence of the combination treatment, the high heterogeneity of the included patients is also the main reason contributing to the negative results of these RCTs (14–17).

Different from other solid tumors, the prognosis and treatment algorithms of HCC are based not only on tumoral characteristics but also on liver function, which leads to high heterogeneity in HCC (18). Currently, the most widely applied model to assess liver functional status is the Child-Turcotte-Pugh (CTP) grade. Nevertheless, the CTP grade was initially established for patients with cirrhosis and portal hypertension undergoing surgery for variceal bleeding rather than for HCC (19, 20). In addition, the CTP grade includes two objective variables: grading of ascites and encephalopathy. Such subjectivity influences the accuracy of the CTP grade for HCC in clinical practice (21).

In 2015, Johnson and colleagues reported a new model, the albumin-bilirubin (ALBI) grade, based solely on albumin and bilirubin, as an alternative method for liver function assessment for HCC (22). Afterward, several studies compared the prognostic performance between the ALBI and CTP grades in HCC cohorts, and the majority of them demonstrated that the ALBI grade performed at least no worse than the CTP grade (23–27). Considering its easy application and objectivity, the ALBI grade has the potential to act as an alternative method to the CTP grade. Notably, the patients included in the negative RCTs about TACE combined with sorafenib for HCC mentioned above were CTP grade A or B7-B8 (14–16). Previous analyses suggested that the ALBI grade performed better than the CTP grade in discriminatory ability for HCC patients with CTP grade A treated with sorafenib monotherapy (25). Therefore, the ALBI grade may have a wider application in clinical research about sorafenib for HCC. Nevertheless, no studies have yet been reported regarding the discriminatory ability comparison between the ALBI and CTP grades for HCC patients treated with TACE combined with sorafenib.

Using artificial intelligence, this study was carried out to compare the prognostic performance associated with overall survival between the ALBI and CTP grades for HCC patients treated with TACE combined with sorafenib.

## Materials and Methods

### Patients’ Criteria

This multicenter retrospective study included patients with HCC treated with TACE combined with sorafenib as the initial treatment between January 2013 and August 2018 at three hospitals. The study was approved by the institutional review boards at the three participating hospitals, and the requirement for informed consent was waived due to its retrospective nature. The study was performed in accordance with the Declaration of Helsinki. The diagnosis of HCC was based on the diagnostic criteria according to the European Association for the Study of the Liver or the American Association for the Study of Liver Diseases (3, 4). Patients from hospital A were regarded as the training cohort, and patients from hospitals B and C were regarded as validation cohorts 1 and 2, respectively.

Patients were included in the study if they met the following criteria: 1) were 18 years or older; 2) had an Eastern Cooperative Oncology Group performance score of 0 or 1; 3) had a definite diagnosis of HCC; 4) were not suitable or unwilling to receive curative treatment such as resection, ablation, or transplantation; 5) had no prior HCC-related treatment; 6) had adequate liver function as follows: CTP grade A or B, with alanine transaminase and aspartate transaminase ≤5 * 3 upper limit of the normal range and total bilirubin ≤5 * 3 upper limit of the normal range; and 7) had adequate renal, clotting, and hematologic function. Patients were excluded if they had any of the following: 1) a contradiction to TACE and sorafenib treatment; 2) infiltrative-type HCC with indistinct borders and a lack of typical enhancement pattern; 3) accompanying or history of any other primary malignancies; and 4) incomplete or missing clinical and follow-up data. Multidisciplinary discussions were carried out pretreatment to decide whether TACE combined with sorafenib was the recommended therapy for the patients. Written informed consent regarding the advantages and disadvantages of the combination treatment, including the potential treatment outcomes, costs, and treatment-related morbidities, was obtained from every included patient.

### Treatment, Assessment, and Calculation of the Liver Function Grades

All patients included in the study underwent TACE combined with sorafenib as an initial HCC-related treatment. All patients underwent conventional TACE, and a detailed description of the procedure has been reported previously (28). The TACE procedure was repeated according to “on demand” mode: when no vital active tumor lesion(s) was observed on contrast-enhanced computed tomography (CT) or magnetic resonance imaging (MRI) 4–6 weeks after the previous procedure, TACE was discontinued, and the patient underwent the next contrast-enhanced CT/MRI and alpha-fetoprotein follow-up every 8–10 weeks; if the contrast-enhanced CT/MRI presented new lesions, the patient was evaluated for repeated TACE (29, 30). The TACE procedures were performed by several interventional radiologists (:_, with 32 years of experience in hospital A, :_ and :_, with 31 and 23 years of experience in hospital B, and :_, with 22 years of experience in hospital C).

Sorafenib (Bayer Healthcare, Leverkusen, Germany) was administered with an initial dose of 400 mg twice daily within 3–7 days after every TACE procedure and was stopped the day before every TACE. Dose reductions to 200 mg twice daily and then 200 mg once daily or temporary interruptions were allowed due to drug-related toxicity. Sorafenib was discontinued in the event of disease progression or unacceptable toxicity.

The primary endpoint of the study was overall survival (OS), defined as the time from the first TACE procedure to any cause of death or the last follow-up (September 1, 2019). The preprocedural ALBI and CTP grades were calculated using the appropriate clinical parameters. In addition, the modified ALBI grade, the platelet-ALBI grade, was also calculated. Details of the calculation of the grades are summarized in Appendix E1.

### Establishment of the Nomograms and Artificial Neural Network in the Training Cohort

The ALBI-based and CTP-based nomograms and ANN model were established based on the independent risk factors associated with OS that were identified by univariate and multivariate analyses in the training cohort. The prognostic performance and discrimination of the nomograms were then validated and compared in the two validation cohorts. For the ANN model, two-thirds of the included patients in the training cohort were randomly included to establish the model, with the remaining one-third used for cross validation (31, 32). A detailed description of the ANN establishment has been reported in our previous studies (33).

### Statistical Analysis

Categorical variables are presented as frequencies and percentages, and continuous variables are presented as medians with 95% confidence intervals (CIs) or means with standard deviations. The baseline characteristics of the three hospitals were compared using *t* test for continuous variables and Fisher’s exact test or the χ^2^ test for categorical variables. OS was estimated using Kaplan-Meier curves. Variables with a P value no more than 0.20 in the univariate analysis were considered strong risk factors associated with OS and were then put into the Cox regression model for multivariate Cox proportional hazards analysis. Variables with P values no more than 0.05 were considered independent risk factors associated with OS. The ALBI-based and CTP-based nomograms were established based on the independent risk factors. The ANN model was established based on the strong risk factors. The prognostic performance and discrimination of the nomograms were validated and compared using the concordance c statistic (C-index), and the significance of the C-index was compared using a Z test. Statistical analyses were performed using SPSS version 22.0 software for Windows (IBM Corporation, Somers, New York), and the nomograms were formulated through the regression modeling strategies package in R language version 3.4.3 software for Windows (R Package for Statistical Computing; www.r-project.org). The ANN model was established using SPSS Clementine version 12.0 software for Windows (IBM Corp, Armonk, New York).

## Results

### Patient Characteristics and the Albumin–Bilirubin and Child–Turcotte–Pugh Grade Comparison

**Figure 1:**
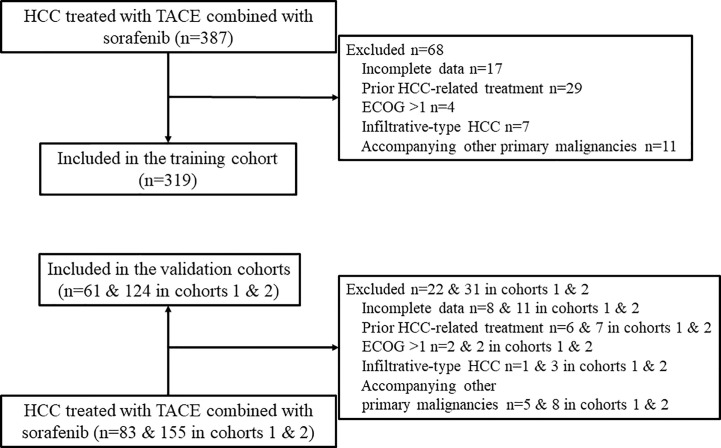
Flow chart of patient selection. HCC, hepatocellular carcinoma; TACE, transarterial chemoembolization; ECOG, Eastern Cooperative Oncology Group.

A total of 504 patients (319, 61, and 124 patients from hospitals A, B, and C, respectively) were included in the study (**Figure 1**). The patients’ baseline characteristics are presented and compared in **Table 1**. The entire median follow-up period was 16.7 months; the median OS was 15.9 months overall, and 15.2, 26.9, and 14.8 months in the training and validation 1 and 2 cohorts, respectively (P = 0.218). The median duration of sorafenib treatment was 13.3 months (range, 1.2–52.6 months). No combination treatment-related death occurred during follow up. Sorafenib was discontinued in 358 (71.0%) patients mainly due to disease progression and intolerable adverse events. Sorafenib-related adverse events occurred in 391 (77.6%) patients, and the most frequent adverse events were hand-foot-skin reaction (51.8%) and diarrhea (39.1%).

**Table 1:** Patient characteristics.

Characteristic	Overall (n = 504)	Training cohortHospital A (n = 319)	Validation cohort		P*
			Hospital B (n = 61)	Hospital C (n = 124)	
Gender					0.134
Male	434 (86.1%)	275 (86.2%)	48 (78.7%)	111 (89.5%)		
Female	70 (13.9%)	44 (13.8%)	13 (21.3%)	13 (10.5%)		
Age (years)					0.031
≤65	430 (85.3%)	281 (88.1%)	52 (85.2%)	97 (78.2%)		
>65	74 (14.7)	38 (11.9%)	9 (14.8%)	27 (21.8%)		
ECOG					<0.001
0	490 (97.2%)	316 (99.1%)	60 (98.4%)	114 (91.9%)		
1	14 (2.8%)	3 (0.9%)	1 (1.6%)	10 (8.1%)		
Hepatitis B (Yes)	422 (83.7%)	271 (85.0%)	51 (83.6%)	100 (80.6%)	0.544
Cirrhosis					0.309
Yes	345 (68.5%)	224 (70.2%)	43 (70.5%)	78 (62.9%)		
No	159 (31.5%)	95 (29.8%)	18 (29.5%)	46 (37.1%)		
ALBI grade					0.501
1	256 (50.8%)	167 (52.4%)	27 (44.3%)	62 (50.0%)		
2	248 (49.2%)	152 (47.6%)	34 (55.7%)	62 (50.0%)		
CTP grade					0.519
A	436 (86.5%)	277 (86.8%)	50 (82.0%)	109 (87.9%)		
B	68 (13.5%)	42 (13.2%)	11 (18.0%)	15 (12.1%)		
BCLC stage					0.048
B	259 (51.4%)	161 (50.5%)	40 (65.6%)	58 (46.8%)		
C	245 (48.6%)	158 (49.5%)	21 (34.4%)	66 (53.2%)		
No. of nodules					0.748
Single	230 (45.6%)	148 (46.4%)	29 (47.5%)	53 (42.7%)		
Multiple	274 (54.4%)	171 (53.6%)	32 (52.5%)	71 (57.3%)		
Maximum tumor size (cm)					0.362
≤5	198 (39.3%)	122 (38.3%)	28 (45.9%)	48 (38.7%)		
5-10	184 (36.5%)	121 (37.9%)	23 (37.7%)	40 (32.3%)		
>10	122 (24.2%)	76 (23.8%)	10 (16.4%)	36 (29.0%)		
AFP (ng/dl)					0.797
≤200	262 (52.0%)	163 (51.1%)	34 (55.7%)	65 (52.4%)		
>200	242 (48.0%)	156 (48.9%)	27 (44.3%)	59 (47.6%)		
AST (U/L)					0.150
≤40	223 (44.2%)	132 (41.4%)	27 (44.3%)	64 (51.6%)		
>40	281 (55.8%)	187 (58.6%)	34 (55.7%)	60 (48.4%)		
ALT (U/L)					0.128
≤40	288 (57.1%)	177 (55.5%)	31 (50.8%)	80 (64.5%)		
>40	216 (42.9%)	142 (44.5%)	30 (49.2%)	44 (35.5%)		
TBIL (μmol/L)					0.036
≤34	468 (92.9%)	303 (95.0%)	53 (86.9%)	112 (90.3%)		
>34	36 (7.1%)	16 (5.0%)	8 (13.1%)	12 (9.7%)		
Albumin (g/L)					0.056
>35	404 (80.2%)	251 (78.7%)	45 (73.8%)	108 (87.1%)		
≤35	100 (19.8%)	68 (21.3%)	16 (26.2%)	16 (12.9%)		

*Chi-square test was used. ALBI, albumin-bilirubin; CTP, Child-Turcotte-Pugh; BCLC, Barcelona Clinic Liver Cancer; AFP, alpha-fetoprotein; AST, aspartate transaminase; ALT, alanine transaminase; TBIL, total bilirubin.

In the entire cohort, 256 and 248 patients were classified as ALBI grade 1 and 2, respectively. There were 436 patients classified as CTP A (311 and 125 patients with A5 and A6, respectively) and 68 patients as CTP B. The details of the ALBI grade and CTP score are presented in **Table 2**. While 224 (87.5%) ALBI grade 1 patients were classified as CTP A5, ALBI grade 2 included 87 (35.1%), 101 (40.7%), and 60 (24.2%) patients with CTP A5, A6, and B, respectively.

**Table 2:** Correspondences between the CTP scores and ALBI grades in the entire cohort (n = 504).

	CTP A5	CTP A6	CTP B
ALBI grade 1	224	24	8
ALBI grade 2	87	101	60

CTP, Child–Turcotte–Pugh; ALBI, albumin-bilirubin.

For patients classified as ALBI grade 1, there was a significantly better survival for CTP A5 compared to CTP A6 and CTP B, with a median OS of 24.5 (18.2–30.8), 14.1 (1.0–27.1), and 5.1 (4.4–5.8) months, respectively (P = 0.038). For patients classified as ALBI grade 2, no significant survival difference for CTP A5, A6, and B was observed, with a median OS of 11.0 (8.3–13.7), 11.8 (9.8–13.8), and 8.0 (5.6–10.3) months, respectively (P = 0.230).

For patients classified as CTP A5, significantly better survival was observed for ALBI grade 1 and 2, with a median OS of 24.5 (18.2–30.8) and 11.0 (8.3–13.7) months, respectively (P < 0.001). Nevertheless, no significant survival difference between ALBI grade 1 and 2 for patients with CTP A6 was observed, with median OS of 14.1 (1.0–27.2) and 11.8 (9.8–13.8) months, respectively (P = 0.154).

### Strong and Independent Risk Factors Associated With Overall Survival in the Training Cohort

**Figure 2:**
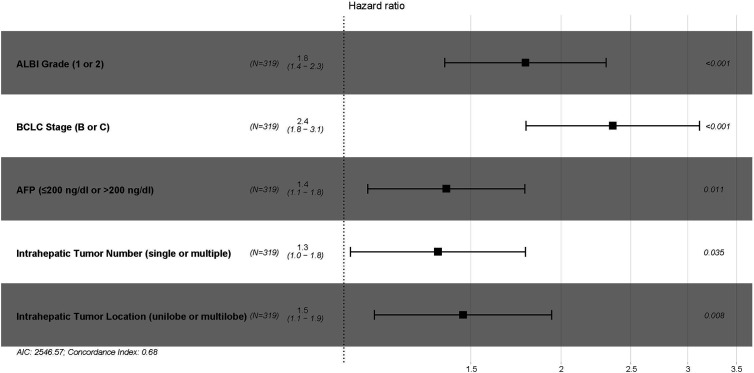
The ALBI-based multivariate Cox proportional hazards regression analysis of risk factors associated with overall survival in the training cohort. ALBI, albumin-bilirubin; BCLC, Barcelona Clinic Liver Cancer; AFP, alpha-fetoprotein.

**Figure 3:**
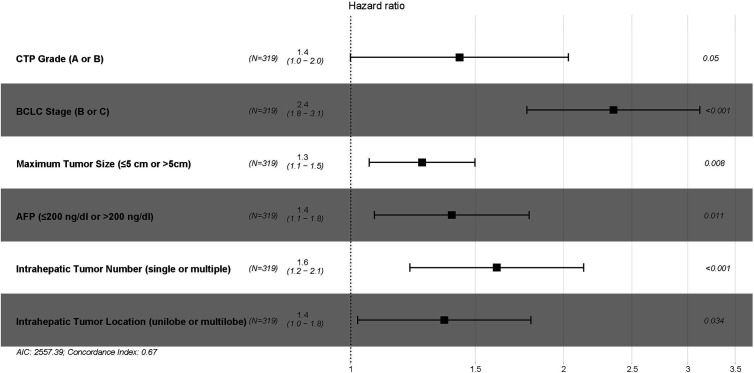
The CTP-based multivariate Cox proportional hazards regression analysis of risk factors associated with overall survival in the training cohort. CTP, Child–Turcotte–Pugh; BCLC, Barcelona Clinic Liver Cancer; AFP, alpha-fetoprotein.

In the training cohort, 7 variables, including ALBI grade, CTP grade, Barcelona Clinic Liver Cancer (BCLC) stage, maximum tumor size (≤5 cm, 5–10 cm, or >10 cm), intrahepatic tumor number (single or multiple), intrahepatic tumor location (unilobe or multilobe), and plasma alpha-fetoprotein (AFP) (≤200 ng/dl or >200 ng/dl), were identified as strong risk factors associated with OS after univariate analysis for the potential variables. The platelet-ALBI grade did not show a significant correlation with OS in the univariate analysis. ALBI-based and CTP-based multivariate analyses were then performed, and both the ALBI and CTP grades were identified as independent risk factors associated with OS. The detailed results are presented in **Figures 2** and **3**.

### Establishment and Validation of the Nomograms

**Figure 4:**
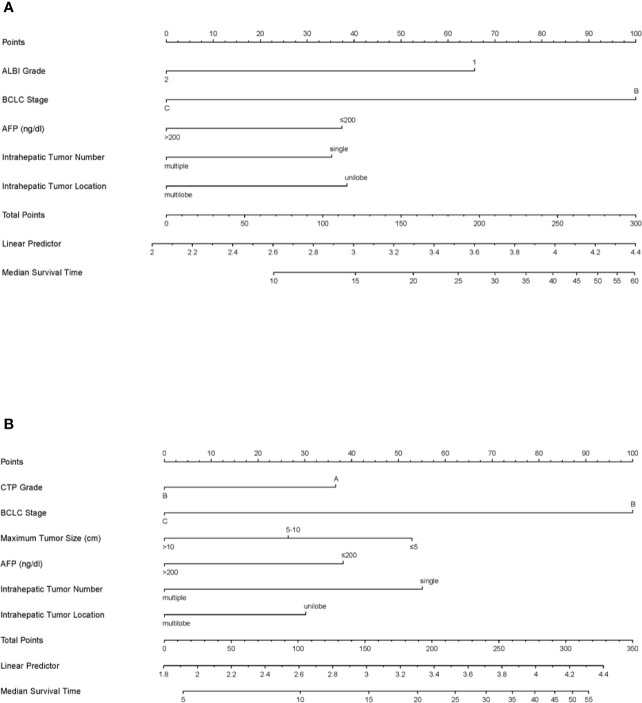
The ALBI-based **(A)** and CTP-based **(B)** nomograms for patients with hepatocellular carcinoma after transarterial chemoembolization combined with sorafenib. ALBI, albumin-bilirubin; BCLC, Barcelona Clinic Liver Cancer; AFP, alpha-fetoprotein; CTP, Child–Turcotte–Pugh.

Based on the results of the multivariate analyses, ALBI-based and CTP-based nomograms were established (**Figure 4**). According to the nomograms, every patient had an ALBI-based grade and a CTP-based grade to predict survival. The prognostic accuracy and discrimination of the two nomograms were then validated and compared in two external validation cohorts. The C-index values of the ALBI-based nomogram and CTP-based nomogram in validation cohort 1 were 0.799 (0.640–0.958) and 0.779 (0.565–0.992), respectively (P = 0.762); they were 0.700 (0.603–0.796) and 0.693 (0.598–0.787) in validation cohort 2, respectively (P = 0.803). Both external validations showed comparable prognostic accuracy and discrimination for these two nomograms.

### Establishment of the Artificial Neural Network Model

**Figure 5:**
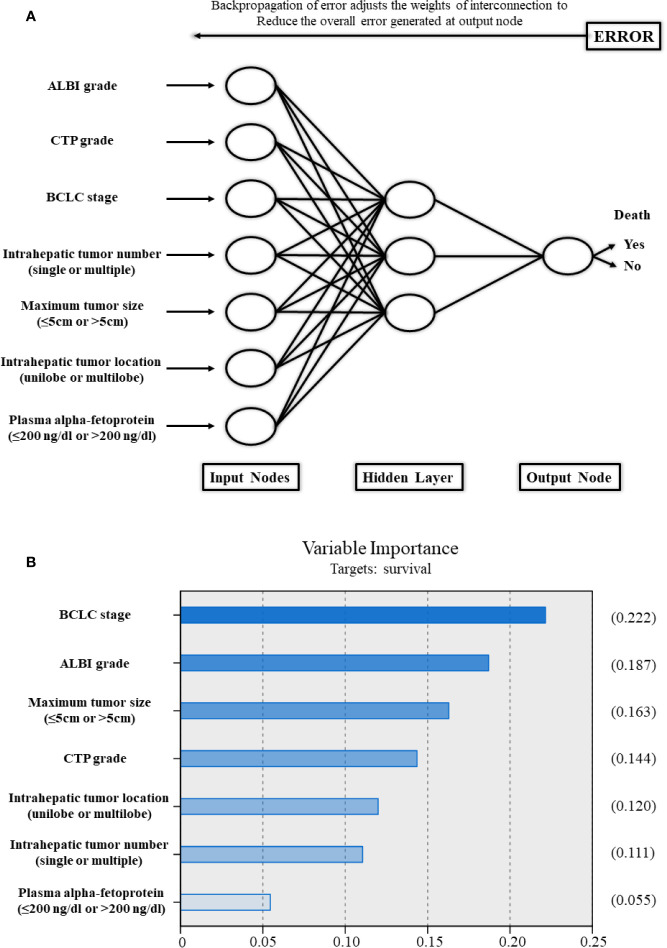
**(A)** Schematic representation of the artificial neural network developed to predict survival for patients with hepatocellular carcinoma after transarterial chemoembolization combined with sorafenib. **(B)** The importance of each variable in the artificial neural network model. ALBI, albumin-bilirubin; CTP, Child–Turcotte–Pugh; BCLC, Barcelona Clinic Liver Cancer.

Based on the strong risk factors mentioned above, an ANN model was established (**Figure 5**). The importance of the risk factors in the ANN model for the BCLC stage, ALBI grade, maximum tumor size (≤5, 5–10, or >10 cm), CTP grade, intrahepatic tumor location (unilobe or multilobe), intrahepatic tumor number (single or multiple), and plasma alpha-fetoprotein (AFP) (≤200 ng/dl or >200 ng/dl) were 0.222, 0.187, 0.163, 0.144, 0.120, 0.111, and 0.055, respectively. In the ANN model, the ALBI grade had higher importance associated with OS compared to the CTP grade.

## Discussion

In this multicenter retrospective series of Chinese HCC patients treated with TACE combined with sorafenib, we demonstrated that the ALBI grade and CTP grade provide comparable prognostic performance using artificial intelligence. The majority of ALBI grade 1 patients were also classified as CTP A5, and a statistically significant difference in median OS was observed between CTP A5, A6, and B for patients with ALBI grade 1.

By combining sorafenib with TACE, a decrease in angiogenesis after the administration of TACE and improved efficacy of TACE should be expected (13). In addition, the treatment effect on liver function may be suppressed by reducing the frequency of TACE through this combination treatment (10). Therefore, the combination treatment should achieve synergetic efficacy for HCC. Nevertheless, the majority of the RCTs (Post-TACE, SPACE, and TACE-2) comparing TACE combined with sorafenib vs. TACE monotherapy demonstrated negative results so far (14–16). Apart from the study design (timing of sorafenib administration in Post-TACE trial, unsuitable scheduled TACE in the SPACE trial, and unsuitable progression assessment in the TACE-2 trial), the heterogeneity of HCC may be another major reason contributing to the negative results of these trials (17). Unlike other solid tumors in which the prognosis is mainly based on the tumor burden, the prognosis of HCC is also influenced by liver function and general status (18).

Currently, the most widely accepted liver function assessment tool for HCC is the CTP grade, which was initially devised to assess the prognosis of patients with cirrhotic-related portal hypertension undergoing surgery for variceal bleeding (19, 20). Nevertheless, several issues should be noted regarding the CTP grade (34). First, the grade includes two subjective variables: grade of ascites and encephalopathy. Second, albumin levels and grade of ascites are inadequate to put together for one grading system, since they are interrelated variables. Third, for the majority of patients, a score of 1 is obtained if converting the prothrombin time into the international normalized ratio. More importantly, for those with CTP A, which is the major inclusion criterion for liver function in many RCTs, the prognosis also varies (25). Therefore, a new assessment tool that overcomes the limitations of the CTP grade mentioned above is warranted for HCC, especially for the study design of HCC-related RCTs.

The ALBI grade was introduced in 2015 by Johnson PJ and colleagues with the aim of overcoming the limitations of the CTP grade by calculating the score based on only two objective variables (serum albumin and bilirubin levels) (22). The prognostic performance of the ALBI grade was then validated and compared in several studies with different kinds of treatments for HCC, with the conclusion that the ALBI grade performed as least no worse than the CTP grade (23–27). Previous studies found that the ALBI grade has the ability to stratify prognosis for patients treated with sorafenib monotherapy in the CTP A group (25). Nevertheless, no effort has been made to explore the performance of the ALBI grade for patients treated with TACE combined with sorafenib. To the best of our knowledge, this study is the first multicenter study comparing the ALBI and CTP grades in a large number of patients treated with TACE combined with sorafenib.

This study demonstrated that the prognostic performance and discrimination of the ALBI grade and the CTP grade were comparable for HCC patients treated with TACE combined with sorafenib. In addition, the ALBI grade has the ability to stratify prognosis for the CTP A group. Notably, the ANN model showed that the importance of the ALBI grade associated with OS was higher than that of the CTP grade, which indicated that the ALBI grade might have a slightly better performance than the CTP grade. Although no significant difference was observed between the ALBI grade and the CTP grade, the easy application and objectiveness of the ALBI grade make it more applicable in large-scale multicenter or international studies. Furthermore, with the ability to select subgroups with better prognosis within the CTP A group, the ALBI grade has the potential to be considered an alternative approach in RCTs, especially for TACE combined with sorafenib for the treatment of HCC.

The interaction among independent risk factors that associated with prognosis in clinical is nonlinear, which makes it relatively inaccurately to distinguish the importance and association with prognosis of each independent risk factor when using conventional linear discriminant analysis (35, 36). ANN has been identified outperform conventional discriminant analysis based on its advances with computer technology application to model a biological neural system structurally and functionally (35, 36). Nomograms are an effective way to predict survival outcomes when the data used to build the nomograms reflect the evolution of the question the nomogram is tasked to answer (37). In this study, the data is retrospectively collected from three academic hospitals in China. Patient and data management is performed in a standard way.

This study has several limitations. First, the retrospective nature of the study may lead to selection bias of the included patients. Nevertheless, the majority of the baseline characteristics of the included patients were not significantly different among the three cohorts. Second, the median OS in validation cohort 1 was longer than that in the other two cohorts. The longer median OS in validation cohort 1 may be due to the better general status and lower tumor burden of these patients compared to those in the other two cohorts. The validation results showed that the prognostic performance of the nomograms was high in validation cohort 1 and comparable with validation cohort 2. Third, considering treatment safety, patients with ALBI grade 3 were excluded. Fourth, we did not analyze other outcomes, such as progression-free survival or the newly introduced time to untreatable progression, that were used in the recently published positive trial about TACE combined with sorafenib for HCC, the TACTICS trial. Finally, and importantly, post-TACE and sorafenib treatment was hard to follow up and analyze; it may be a risk factor associated with survival. Nevertheless, even well-designed RCTs are also hard to manage post-treatment after targeted therapy. Further studies with well-designed protocols and detailed data on this topic are warranted.

In conclusion, the ALBI grade and CTP grade have comparable prognostic performance and discrimination for HCC patients treated with TACE combined with sorafenib. Considering its objectiveness and easy application, the ALBI grade has the potential to be an alternative for liver function assessment tool. ALBI grades 1 and 2 have the potential to serve as stratification factors for clinical trials of TACE combined with systemic therapy. Additional well-designed studies with more detailed information are warranted.

## Data Availability Statement

The raw data supporting the conclusions of this manuscript will be made available by the authors, without undue reservation, to any qualified researcher.

## Ethics Statement

The study was approved by the institutional review boards at the three participating hospitals, and the requirement for informed consent was waived due to its retrospective nature. The study was performed in accordance with the Declaration of Helsinki. The studies involving human participants were reviewed and approved by The First Affiliated Hospital of Soochow University. The ethics committee waived the requirement of written informed consent for participation.

## Author Contributions

C-FN, X-LZ, B-YZ, Z-PY, J-HS, LZ, W-SW, and ZL contributed to the study concept and design. LZ, Z-HH, M-JY, G-HZ and SZ contributed to the acquisition of clinical data. B-YZ contributed to the statistical analysis. B-YZ wrote the first draft of the manuscript. C-FN and X-LZ supervised and oversaw the study. All authors contributed to the article and approved the submitted version.

## Funding

This study was supported by the National Natural Science Foundation of China (81901847, 81771945, and 81971713), the Jiangsu Medical Innovation Team (CXTDB2017006), the Natural Science Foundation of Jiangsu Province (BK20190177), the Natural Science Foundation of Zhejiang Province (LZ18H180001), the Suzhou Science and Technology Youth Plan (KJXW2018003) and “Six One Projects” for High-Level Health Personnel in Jiangsu Province (LGY2018077). Funding source had no involvement in the financial support for the conduct of the research and preparation of the article.

## Conflict of Interest

The authors declare that the research was conducted in the absence of any commercial or financial relationships that could be construed as a potential conflict of interest.

The reviewer JJ declared a past co-authorship with several of the authors B-YZ, J-HS, G-HZ and C-FN to the handling editor.

## Abbreviations

HCC, hepatocellular carcinoma; TACE, transarterial chemoembolization; ALBI, albumin-bilirubin; CTP, Child-Turcotte-Pugh; OS, overall survival; ANN, artificial neural network; VEGF, vascular endothelial growth factor; PDGF, platelet-derived growth factor; RCTs, randomized controlled trials; CT, computed tomography; MRI, magnetic resonance imaging; ANN, artificial neural network; CI, confidence interval; BCLC, Barcelona Clinic Liver Cancer; AFP, alpha-fetoprotein.
