# Supplementary material for: Prognostic Performance of Albumin–Bilirubin Grade With Artificial Intelligence for Hepatocellular Carcinoma Treated With Transarterial Chemoembolization Combined With Sorafenib
Source: Front Oncol. 2020 Dec 18;10:525461. doi: 10.3389/fonc.2020.525461 (PMC7775577; doi:10.3389/fonc.2020.525461)
Supplement: Supplementary file 1 [file Table_1.docx]

**Appendix E1: Calculation and grade of the ALBI, CTP, and PALBI scores**

The ALBI score was calculated as −0.085 × (albumin g/L) + 0.66 × log10 (total bilirubin umol/L). The ALBI was classified into three grades: grade 1: ≤−2.6, grade 2: >−2.6, ≤−1.39, and grade 3: >−1.39. The CTP score was calculated using total bilirubin, albumin, prothrombin time, and the clinical findings of encephalopathy and ascites. The CTP score was defined as follows: grade A, 5–6 points and grade B, 7–9 points. The PALBI score was calculated as 2.02 × log10 (total bilirubin umol/L) – 0.37 × [log10 (total bilirubin umol/L)]2 – 0.04 × (albumin g/L) – 3.48 × log10 (platelets 1000/μL) + 1.01 × [log10 (platelets 1000/μL)]2. The PALBI was classified in three grades: grade 1: ≤−2.53, grade 2: >−2.53, ≤−2.09, and grade 3: >−2.09.
